# Elevated Plasma Levels of 3-Hydroxyisobutyric Acid Are Associated With Incident Type 2 Diabetes

**DOI:** 10.1016/j.ebiom.2017.12.008

**Published:** 2017-12-07

**Authors:** Adil Mardinoglu, Silvia Gogg, Luca A. Lotta, Alena Stančáková, Annika Nerstedt, Jan Boren, Matthias Blüher, Ele Ferrannini, Claudia Langenberg, Nicholas J. Wareham, Markku Laakso, Ulf Smith

**Affiliations:** a Science for Life Laboratory, KTH - Royal Institute of Technology, Stockholm, Sweden; b Department of Biology and Biological Engineering, Chalmers University of Technology, Gothenburg, Sweden; c Department of Molecular and Clinical Medicine, University of Gothenburg, Sahlgrenska University Hospital, Gothenburg, Sweden; d MRC Epidemiology Unit, University of Cambridge, Cambridge, UK; e Institute of Clinical Medicine, Internal Medicine, University of Eastern Finland, Kuopio University Hospital, Kuopio, Finland; f University of Leipzig, Department of Medicine, Leipzig, Germany; g CNR Institute of Clinical Physiology, Pisa, Italy

**Keywords:** 3-Hydroxyisobutyric acid (3-HIB), Branched-chain amino acids (BCAAs), T2D, Insulin resistance, Insulin secretion

## Abstract

Branched-chain amino acids (BCAAs) metabolite, 3-Hydroxyisobutyric acid (3-HIB) has been identified as a secreted mediator of endothelial cell fatty acid transport and insulin resistance (IR) using animal models. To identify if 3-HIB is a marker of human IR and future risk of developing Type 2 diabetes (T2D), we measured plasma levels of 3-HIB and associated metabolites in around 10,000 extensively phenotyped individuals. The levels of 3-HIB were increased in obesity but not robustly associated with degree of IR after adjusting for BMI. Nevertheless, also after adjusting for obesity and plasma BCAA, 3-HIB levels were associated with future risk of incident T2D. We also examined the effect of 3-HIB on fatty acid uptake in human cells and found that both HUVEC and human cardiac endothelial cells respond to 3-HIB whereas human adipose tissue-derived endothelial cells do not respond to 3-HIB. In conclusion, we found that increased plasma level of 3-HIB is a marker of future risk of T2D and 3-HIB may be important for the regulation of metabolic flexibility in heart and muscles.

## Highlights

• The plasma levels of 3-HIB are associated with future risk of incident T2D.
• 3-HIB levels are not robustly associated with degree of IR after adjusting for BMI.
• 3-HIB levels affect the fatty acid uptake in both HUVEC and human cardiac endothelial cells.
• 3-HIB levels do not affect the fatty acid uptake in human adipose tissue-derived endothelial cells.

## 1 Introduction

Insulin resistance (IR) is a common consequence of obesity and the major driver of the current global epidemic of type 2 diabetes (T2D) and associated diseases. However, not all obese individuals develop IR or T2D. Thus, we need reliable and easily measured biomarkers of IR and risk of future disease in order to initiate early clinical interventions (Mardinoglu and Nielsen, 2012). IR does not only include fat and carbohydrate metabolism but is also associated with amino acid metabolism. Circulating levels of branched-chain amino acids (BCAAs), including valine, leucine and isoleucine, are elevated in obese subjects and a distinctive metabolic signature related to BCAA catabolism has been revealed through metabolomics profiling of obese versus lean subjects (Newgard et al., 2009). Levels of BCAAs as well as aromatic amino acids, including tyrosine and phenylalanine, also predict future development of T2D independent of age, sex, body mass index (BMI) and family history (Wang et al., 2011). However, mechanistic explanations for how increased BCAAs can cause insulin resistant states remain unclear.

**Fig. 1:**
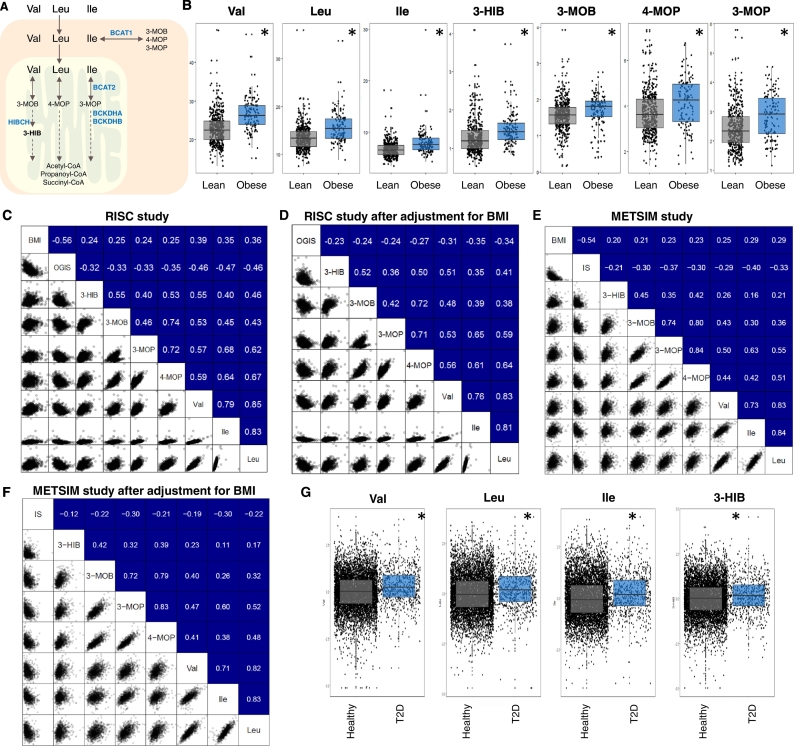
Plasma levels of 3-HIB are strongly related to BMI. A) Catabolism of branched-chain amino acids (BCAAs) including valine (val), leucine (leu) and isoleucine (Ile). B) Plasma levels of metabolites involved in BCAA catabolism in lean and obese subjects in RISC study. The Spearmen correlation of BMI, OGIS and the plasma levels of metabolites involved in BCAA metabolism before (C) and after eliminating the effect of BMI (D) in the RISC study. Correlation of BMI, IS and the plasma levels of metabolites involved in BCAA metabolism before (E) and after eliminating the effect of BMI (F) in the METSIM study. G) The plasma level of the metabolites involved in BCAA metabolism in healthy subjects and T2D patients in the EPIC-Norfolk study. (*P-value < 0.05).

A previous study by Jang et al. (2016) has identified 3-hydroxyisobutyrate (3-HIB), a valine metabolite and derived from 3-hydroxyisobutyryl-coenzyme A by HIBC hydrolase (encoded by *Hibch*) (Fig. 1A) as a paracrine regulator of trans-endothelial fatty acid transport in the skeletal muscle in mice. The authors showed that 3-HIB, secreted from muscle cells, enhances their fatty acid uptake leading to increased lipid accumulation and induction of whole-body IR. Inhibiting synthesis of 3-HIB reduced fatty acid uptake by skeletal muscle microvascular endothelial cells and prevented IR.

However, before concluding that 3-HIB also can contribute to human disease, the findings need to be validated in man. The human translation of the experimental data in mice was less convincing. 3-HIB levels were elevated in the muscle of diabetic db/db mice but only in three of the seventeen analyzed human T2D subjects compared to non-diabetic individuals (Jang et al., 2016). Furthermore, a positive effect of 3-HIB on fatty acid uptake in human cells was only shown in HUVEC cells which are of macrovascular, rather than tissue-related microvascular origin. Furthermore, to be of clinical predictive value we need to understand if plasma levels of 3-HIB can be used as a risk indicator of IR and future development of T2D in man. If so, *Hibch* can be a target for developing efficient treatment strategies for T2D. To examine this, we analyzed 3-HIB plasma levels in around 10,000 human subjects and their relation to future development of T2D. We also examined the effect of 3-HIB on fatty acid uptake by human microvascular endothelial cells from the adipose tissue and heart as well as by HUVEC macrovascular cells.

## 2 Materials and Methods

### 2.1 Plasma Metabolomics Data

The subjects included in this study have been described previously (Lee et al., 2016, Mardinoglu et al., 2017). Signed informed consent has been obtained from human subjects in all cohorts, and the study protocol has been approved by the relevant ethical committees. The studies were conducted according to standards indicated by the Declaration of Helsinki.

Measurement of plasma levels of metabolites involved in BCAA metabolism was performed using LC-MS. Briefly, the liquid chromatography-tandem mass spectrometry (LC-MS/MS) platform was based on a Waters ACQUITY ultraperformance liquid chromatography (UPLC) system and a Thermo-Finnigan LTQ mass spectrometer operated at nominal mass resolution, which was equipped with an electrospray ionization (ESI) source and a linear ion trap (LIT) mass analyzer. The sample extract was dried and then reconstituted in acidic or basic LC-compatible solvents, each of which contained 12 or more injection standards at fixed concentrations.

One aliquot was analyzed using acidic positive ion-optimized conditions and the other was analyzed using basic negative ion-optimized conditions in two independent injections using separate dedicated columns (Waters UPLC BEH C18-2.1 × 100 mm, 1.7 lm). Extracts reconstituted in acidic conditions were gradient eluted using water and methanol containing 0.1% formic acid, whereas the basic extracts, which were also eluted using water/methanol, contained 6.5 mM ammonium bicarbonate. The MS analysis alternated between MS and data-dependent MS/MS scans using dynamic exclusion, and the scan range was from 80 to 1000 *m*/*z*. Following log transformation, Welch's two-sample *t*-test was used to identify levels of BCAA metabolites that differed significantly between the biological samples.

### 2.2 Fatty Acid Transport in Human Adipose Tissue-and Cardiac-Derived Endothelial Cells

Fatty acid uptake was measured using the Quencher-Based Technology (QBT) Fatty Acid Uptake Assay Kit (Molecular Devices) as reported (Liao et al., 2005). Briefly, human adipose tissue– and cardiac-derived microvascular (c-MVEC) (LONZA-CC-7030) or HUVEC (ATCC-CRL-1730) macrovascular endothelial cells were plated onto a 96-well black-wall/clear-bottom plate at 10,000 cells/well in endothelial cell culture media EBM-2. After 20 h, the cells were stimulated with no additions, 3-HIB (5 mM) or OA (300uM) for 24 h. Cells were then plasma-deprived for 1 h (3-HIB and OA still included), followed by the addition of QBT Fatty loading buffer to each well. Kinetic readings were started immediately with a Tecan Infinite M200 fluorescence plate reader.

### 2.3 Sampling and Statistical Analysis

Prospective association with incident type 2 diabetes of BCAA-pathway metabolites were performed in up to 6991 individuals from the population-based EPIC-Norfolk study, including 693 incident cases of T2D and 6298 non-cases. EPIC-Norfolk is a prospective cohort study of over 25,000 individuals living in the Norfolk County in East Anglia (United Kingdom) at recruitment in 1993–1997 (Day et al., 1999). In EPIC-Norfolk, BCAA-related metabolites were measured using an untargeted UPLC-MS/MS platform (DiscoveryHD4® platform - Metabolon Inc.) in citrated plasma samples collected at baseline as previously described (Lotta et al., 2016). Before analysis, metabolite levels reported by Metabolon were ln-transformed, winsorised to 5 SDs and standardised to a mean of 0 and a standard deviation of 1. Cox proportional hazards regression with Prentice weighting and robust standard errors was used to estimate multivariable-adjusted hazard ratios (HRs) for incident type 2 diabetes and their 95% confidence intervals (CI). The levels of blood metabolites at baseline were the exposure variable in the analysis. Age was used as the underlying time scale from recruitment to study exit (diagnosis of type 2 diabetes or censoring) of each participant. Covariates in the association analysis included age at baseline, sex, experimental batch, BMI, waist circumference, educational attainment, family history of type 2 diabetes, smoking status, alcohol consumption and self-reported level of physical activity.

Analyses were conducted using R (https://cran.r-project.org/) and STATA v14.2 (StataCorp, College Station, Texas 77845 USA).

## 3 Results

### 3.1 BMI Is a Major Regulator of Plasma BCAA Metabolite Levels

We analyzed the plasma levels BCAAs, branched-chain ketoacids (BCKAs) including 3-Methyl-2-oxobutyric acid (3-MOB), 3-Methyl-2-oxopentanoic acid (3-MOP) and 4-Methyl-2-oxopentanoic acid (4-MOP) as well as 3-HIB in up to 10,000 individuals available to the IMI/EMIF collaborative study. First, we analyzed plasma of 955 subjects involved in the Relationship between Insulin Sensitivity and Cardiovascular Disease (RISC) Study (Hills et al., 2004) using the validated oral glucose insulin sensitivity (OGIS) index (Mari et al., 2001) as a marker of glucose clearance. We measured the plasma levels of these metabolites in lean (BMI < 25) and obese (BMI > 30) subjects and found that the levels of 3-HIB as well as the other measured BCAA metabolites were significantly (P-value < 0.05) upregulated in obese compared to lean subjects (Fig. 1B). Spearman's correlation coefficients (*r*) between the BMI, OGIS and plasma levels of these metabolites also showed significant (P-value < 0.05) correlations between each other (Fig. 1C). We also performed partial correlation analysis and found the correlations between the plasma levels of 3-HIB and OGIS to be markedly weakened by adjusting for BMI (Fig. 1D).

To further validate these data, we examined the BCAA metabolites in plasma samples from randomly selected 992 non-diabetic individuals who participated in a 6-year follow-up in the cross-sectional population-based METabolic Syndrome In Men (METSIM) Study (Stancakova et al., 2009). Subjects involved in the METSIM study were extensively phenotyped and the validated Matsuda insulin sensitivity index (ISI) (Matsuda and DeFronzo, 1999) was used as a marker of insulin sensitivity (IS). We measured the plasma levels of 3-HIB as well as the other BCAA metabolites and found the plasma levels significantly correlated with BMI and the Matsuda ISI (Fig. 1E). After adjusting for the effect of BMI, the correlation between the plasma levels of 3-HIB and the Matsuda ISI was weakened and became borderline significant (Fig. 1F). In both of these European cohorts, we found that OGIS and Matsuda ISI had significantly higher correlations with valine, which is catabolized to 3-HIB, compared to the correlations with 3-HIB both before and after adjusting for the effect of BMI. Taken together, these results support 3-HIB as a marker of BMI and associated degree of IS.

### 3.2 Association of BCAA Metabolite Levels With Incident T2D

It has been shown that circulating levels of BCAAs are associated with risk of future T2D in non-diabetic individuals from the general population and that genetic risk markers for T2D also identify individuals with elevated BCAA levels (Lotta et al., 2016, Xu et al., 2013). In this context, we studied the association of baseline plasma 3-HIB levels with incident T2D in up to 6991 individuals from the population-based EPIC-Norfolk study (including 693 incident cases of T2D and 6298 non-cases) using weighted Cox regression. Baseline 3-HIB levels were indeed associated with incident T2D, but the association was greatly attenuated after adjusting for BMI and waist circumference (WC): a marker of abdominal obesity (Table 1). The association was further attenuated after adjusting for baseline BCAAs levels (Table 1).

**Table 1:**
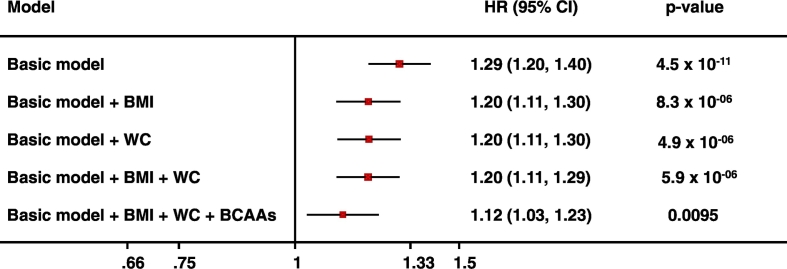
Association of baseline 3-HIB levels with incident T2D in the EPIC-Norfolk study. In Basic model, the adjustment was made for study specific covariates including age at baseline, experimental batch, BMI, waist circumference, educational attainment, family history of type 2 diabetes, smoking status, alcohol consumption and self-reported level of physical activity to account for confounding effects of other risk factors of T2D.

Plasma levels of 3-HIB were significantly (P-value < 0.05) increased in subjects with T2D and so are the levels of other measured BCAA metabolites (Fig. 1H). In order to understand if the level of 3-HIB is dependent on the level of valine in subjects with T2D, we calculated the correlation between the levels of these two BCAA metabolites and observed a significant (P-value < 0.05) correlation (*r* = 0.36) in the EPIC-Norfolk study. Similarly, we calculated the correlations between the levels of 3-HIB and valine, leucine and isoleucine in the METSIM study and found significantly higher correlations between 3-HIB and valine (*r* = 0.262) compared to leucine (*r* = 0.213) and isoleucine (*r* = 0.156). Taken together, we conclude that 3-HIB plasma levels are increased in subjects with T2D, like other BCAAs, and 3-HIB plasma levels are associated with future development of incident T2D. However, the association with IR in man is confounded by the association with BMI and, thus, amount of adipose tissue.

### 3.3 3-HIB Does Not Enhance Fatty Acid Transport in Adipose Tissue-Derived Endothelial Cells

**Fig. 2:**
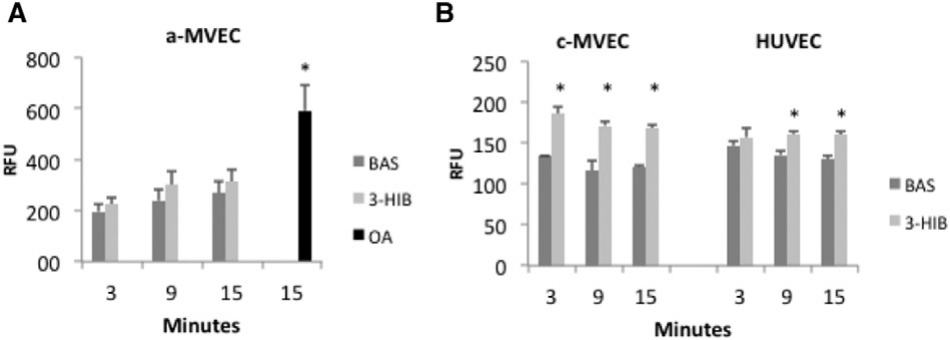
Fatty acid transport in human microvascular and cardiac-derived endothelial cells. A) Fatty acid transport in human adipose tissue-derived microvascular endothelial cells show no effect of preincubating the cells for up to 24 h with 5 mM 3-HIB while preincubation with oleic acid (OA) (300 μM) increased fatty acid uptake. B) However, fatty acid uptake by cardiac-derived endothelial cells was significantly increased by 3-HIB.

Since BMI and amount of adipose tissue were clearly correlated with 3-HIB levels, we asked if the adipose tissue is a target for 3-HIB to increase fatty acid uptake and flux as shown in mouse skeletal muscle and HUVEC cells by Jang et al. (2016). We pre-incubated human adipose tissue-derived microvascular endothelial cells with concentrations of 3-HIB used by Jang et al. (2016) for up to 24 h but saw no increase in fatty acid uptake while oleic acid, as expected, increased uptake (Fig. 2A). However, both human HUVEC and cardiac microvascular endothelial cells responded to 3-HIB with increased fatty acid uptake (Fig. 2B). Thus, 3-HIB is not a uniform enhancer of peripheral tissue fatty acid uptake in human endothelial cells but the effect in cardiac-derived endothelial cells indicates that 3-HIB can be involved in regulating both skeletal and heart muscle fatty acid uptake which can make it an interesting metabolic target of peripheral lipotoxicity in man.

## 4 Discussion

From a clinical point of view, it is important to early identify biomarkers in man which are associated with risk of developing future disease. This allows the implementation of intervention procedures and can prevent the development of incident disease. In a previous publication, Jang et al. (2016) showed that 3-HIB is secreted by mouse skeletal muscles genetically engineered to increase PGC1 expression and that 3-HIB increases skeletal muscle fatty acid uptake and induces insulin resistance in mice. We here asked if plasma levels of 3-HIB is a potential candidate for identifying future risk of T2D. Our study documented the importance of having access to large prospective databases of individuals with extensively characterized phenotypes and with clinical outcome data in order to evaluate the importance of potential novel biomarkers and mechanisms of disease.

Our results showed that plasma 3-HIB levels are increased in individuals with IR but also that obesity is an important confounding factor for this relationship. Indeed, elevated 3-HIB levels were associated with future development of T2D, but this risk was markedly reduced when BMI was taken into account. Thus, our data in man showed that plasma levels of 3-HIB are strongly influenced by not only skeletal muscle but also by amount of adipose tissue. This was further supported by the data where 3-HIB-associated degree of insulin sensitivity was of less prominence when BMI was accounted for. In fact, recent studies have shown that activity of BCAA metabolism is high in the adipose tissue, actually higher than in skeletal muscles per unit weight in mice (Herman et al., 2010). It has also been shown that obesity leads to decreased catabolism of BCAAs in the adipose tissue in man although this has been questioned (Lackey et al., 2013, Mardinoglu et al., 2014). Important data in this respect come from studies in monozygotic twins discordant for obesity showing increased plasma levels of BCAA in the obese compared to the non-obese twin. The obese twin had lower mitochondrial DNA copy number as well as downregulated levels of key markers of BCAA oxidation in the adipose tissue (Pietilainen et al., 2008). These data, together with our current findings in very large human cohorts, raise the importance of the adipose tissue for BCAA metabolism and plasma 3-HIB levels.

To examine if 3-HIB also regulates fatty acid transport in human adipose tissue, we examined its effect in human adipose tissue-derived endothelial cells but saw no such effect. However, similar to the data by Jang et al. (2016), we also found positive effects of 3-HIB on fatty acid transport in human HUVEC cells. Interestingly, 3-HIB also increased fatty acid transport in human cardiac-derived endothelial cells which suggests that both skeletal muscle and heart fatty acid transport are under regulation of 3-HIB. The fact that 3-HIB did not increase uptake in the adipose tissue but did in muscle/heart endothelial cells raises the question if 3-HIB may be a regulator of metabolic flexibility in muscle tissues in man and where increased fatty acid uptake is associated with reduced carbohydrate metabolism. Such an effect would then also be important for the development of whole-body IR and reduced peripheral glucose uptake.

A major strength of this study is the access to large European human cohorts with extensive phenotyping and with prospective follow-up to identify future development of incident disease. This is accomplished by the EU/IMI-funded large collaborative project EMIF where one focus is to identify novel markers of obesity-associated metabolic complications. A weakness of the study is that the databases only include European cohorts and validation of these data in other ethnic groups is required.

Taken together, 3-HIB is likely to be released by both skeletal muscles and the adipose tissue in man and elevated levels reflect the compound effect of these tissues. However, 3-HIB is also marker of future risk of T2D and may be important for the regulation of metabolic flexibility in heart and muscles.

## Conflicts of Interest

The authors declare no competing interests.

## Author Contributions

AM, LAL and AS analyzed the data. JB, MB, EF, CL, NJW, ML and US were involved in the generation of metabolomics data. SG, AN and US generated data related to the effect of 3-HIB on different cell types. AM and US wrote the paper and all authors were involved in editing the paper.
